# Recurrent urethral obstruction secondary to large vulval inclusion cyst: a remote complication of female genital mutilation: a case report

**DOI:** 10.1186/s12905-023-02653-0

**Published:** 2023-09-18

**Authors:** AbdelAziem A. Ali, Israa Badreldin

**Affiliations:** 1 Department of Obstetrics and Gynecology, Faculty of Medicine, Kassala University, P.O. Box 496, Kassala, Sudan; 2 Alshuhada Teaching Hospital, Khartoum, Sudan

**Keywords:** Female, Genital mutilation, Inclusion cyst, Urine retention

## Abstract

**Background:**

Female genital mutilation/cutting (FGM/C) is a major public health problem, particularly in developing countries.

**Case presentation:**

The authors reported a case of 48-year old multiparous woman presented to Kassala Hospital, east Sudan, with recurrent urine retention resulting from urethral obstruction, which was caused by large vulval inclusion cyst. A traditional birth attendant circumcised her when she was 5 year old. Five years before her presentation the patient observed a painless swelling in her vulva, which was gradually increasing in size. She presented to the hospital with urine retention seeking medical care. Local examination showed a large cystic swelling originating in the circumcision line and covering the introitus. A diagnosis of inclusion cyst at the site of circumcision was made. The cyst was large enough causing bladder outlet obstruction and when the patient advised to tilt it away from the urethral orifice she passed urine without difficulties. The cyst was surgically removed by dissection along the lines of cleavage, which measured 10 × 9.2 cm and weighed 1.2 kg.

**Conclusion:**

This case report indicates that FGM is a serious public health problem and there should an urgent intervention such as planned health education campaigns to end FGM practice.

## Background

Female genital mutilation/cutting (FGM/C) is a major public health problem, particularly in developing countries [1]. The practice is considered as a violation of the human rights of girls and women [2]. Over 200 million women and girls have undergone FGM/Cutting and live with its complications all over the world [3]. Female genital mutilation is highly prevalent in Sudan, it is reported in 83.3% among school girls and many serious FGM- related health complications have been reported, Table 1 [4]. In Sudan almost all, the midwives in our community are practicing FGM, they have very low level of awareness regarding the different types of the FGM [5]. The majority of these midwives not view the practice as harmful and insisted to continue in practicing it for cultural reason [5]. Most of the literature reported on obstetricians, gynecologists and other health professionals dealing with the victims of FGM/Cutting. There is knowledge gaps among different health care providers including pediatricians, general practitioners and community health workers, which might represent challenges concerning the attitudes towards FGM/Cutting [6].

**Table 1:** Reported complications of FGM/Cutting

Immediate Complications	Remote Complications
Pain	Pelvic inflammatory disease
Haemorrahe	Pelvic infections
Genital swelling	Chronic pelvic pain
Rectal injury	Menstrual problems
Infection/ Necrotizing Fasciitis	Vulvodynia
Shock	Inclusion cyst
Urine retention	Recurrent urinary tract infection
Dysuria	Keloid formation
Psychological problems	Sexual problems

## Case presentation

**Fig. 1:**
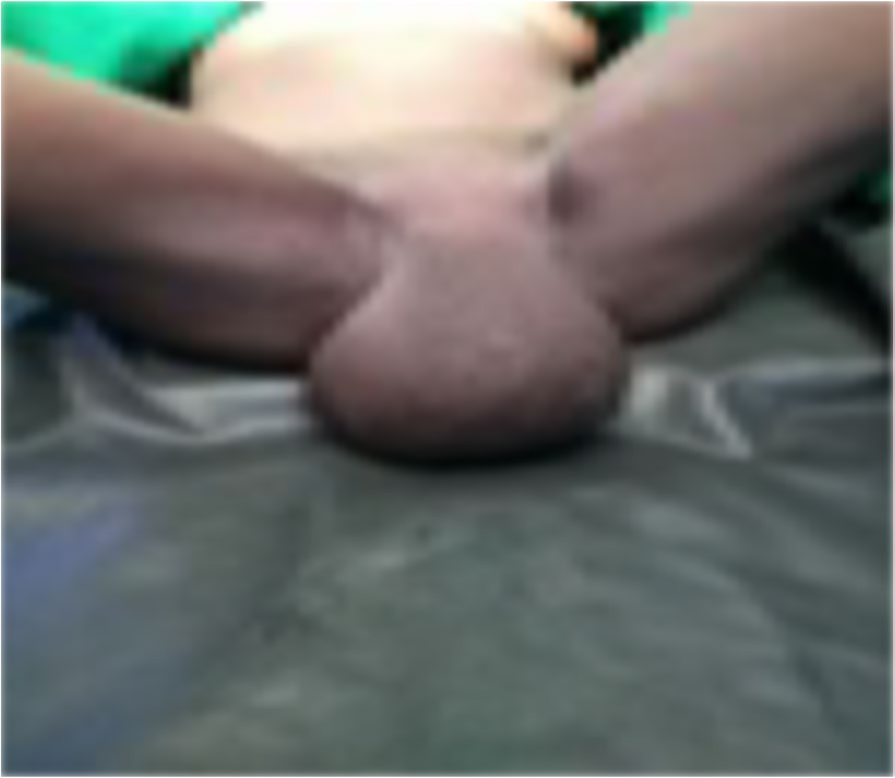
Large inclusion cyst complicating urine retention

**Fig. 2:**
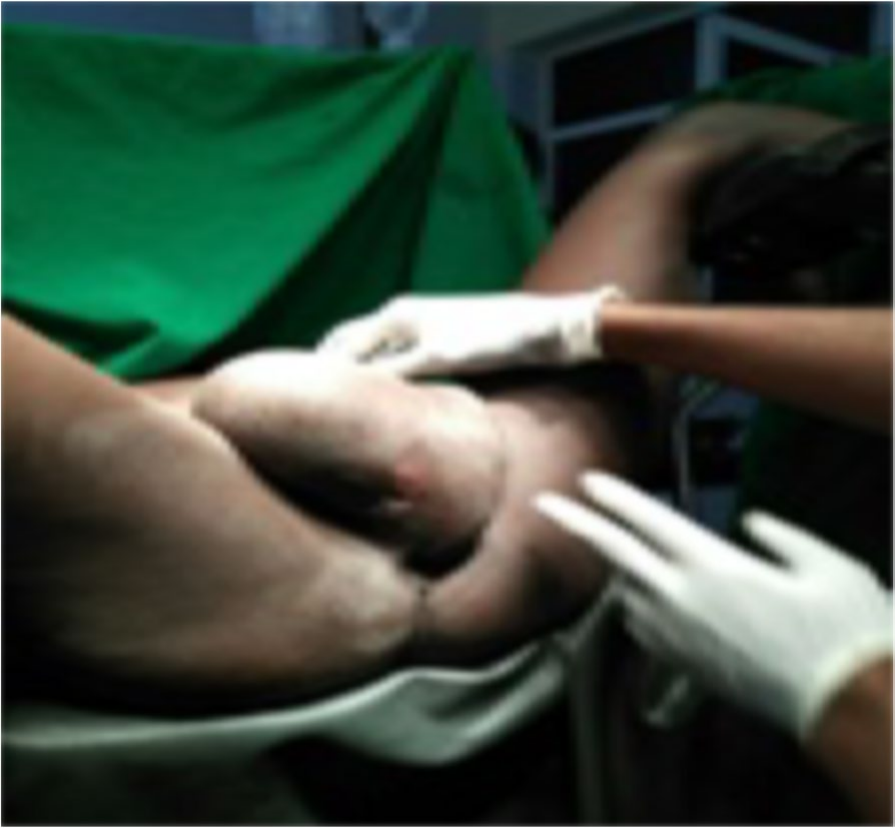
Large inclusion cyst covering the introitus

**Fig. 3:**
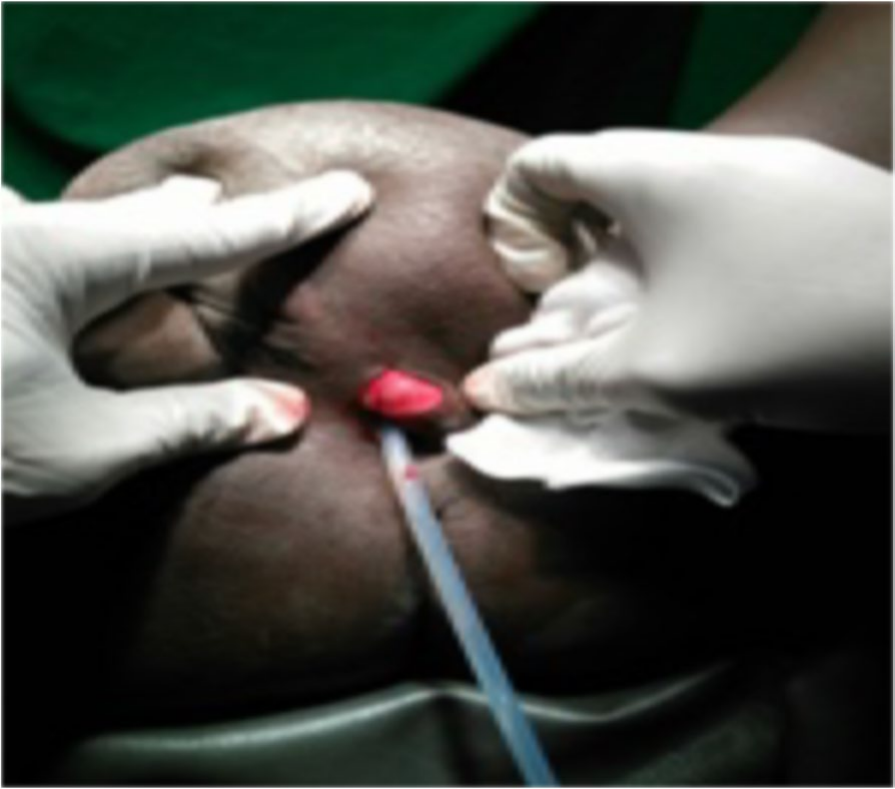
Large inclusion cyst: Incision is made to insert Foley catheter

The authors reported a case of 48-year old multiparous woman presented to Kassala Hospital, east Sudan, with recurrent urine retention resulting from urethral obstruction, which was caused by large vulval inclusion cyst. She had obstetric history of spontaneous home vaginal deliveries, the last one seven year back. A traditional birth attendant circumcised her when she was 5 year old. De-infibulation, re-infibulation and episiotomy were performed after each delivery. Five years before her presentation our patient observed a painless swelling in her vulva, which was gradually increasing in size. She neglected it since a relative midwife had re assured her. The swelling enlarged to extent that made the sexual intercourse very difficult. In the last month prior to her presentation, she suffered urine retention three times for which the nearby health care provider relieved her symptom by temporary catheterization of her urinary bladder without further investigation and treatment. She presented to the hospital with the fourth time of urine retention seeking medical care and asking for the cause. Local examination showed a large cystic swelling originating in the circumcision line and covering the introitus (Fig. 1). A diagnosis of inclusion cyst at the site of circumcision was made. Systemic and bimanual pelvic examination was unremarkable. Both kidneys and other abdominal organs appeared normal on sonogram. The cyst was large enough causing bladder outlet obstruction and when the patient advised to tilt it away from the urethral orifice she passed urine without difficulties (Fig. 2). Her urine analysis showed uncountable pus cell with trace of protein. After inserting an indwelling Foley catheter (Fig. 3), the cyst was excised under spinal anesthesia through a vertical incision in the skin. The cyst was surgically removed by dissection along the lines of cleavage. The cyst measured 10 × 9.2 cm and weighed 1.2 kg. The redundant skin was excised and the edges were approximated. There were no complications following surgery and the patient was discharged on day two following the surgery because she resided in a remote site from the hospital. The patient followed for six months after she had been discharged home and she was symptom free.

## Discussion

Female genital mutilation is a common procedure in developing countries that causes early and late complications. This case indicates the seriousness of FGM and it is severe enough to cause urine retention. In women, this condition, bladder outlet obstruction (BOO), remains a poorly understood condition and is much rarer in comparison with male. This cyst enlarged until it created re angulation of the urethra in the urinary bladder and thus a blockage at its base. In consistent with our report, *Ramart* reported BOO in 50 years old woman presented with a huge introitus mass [7]. Urethral obstruction is a life-threatening condition and complete urethral obstruction in a patient for only few days can be fatal due to renal failure and retention of metabolic wastes [8, 9]. There have been reports on deaths attributed to FGM, from tetanus in Nigeria, a country that share Sudan similar social and cultural characteristics [10]. Female genital mutilation is a dangerous practice that leads to many serious complications [11]. Immediate health complications of FGM include hemorrhage, sepsis, severe pain and psychological consequences, while remote complications such as keloid formation, sexual dysfunction, primary infertility and obstetric complications are also reported [12]. In Sudan and to achieve demedicalization of FGM, a national campaign aiming to fight FGM by raising awareness among urban and rural communities was done by trained medical students who approached women, men, high school students, doctors, midwives and other health care professional in their small communities [13]. *Dirie* and *Lindmark* reported urinary retention, urinary tract infection, and urinary problems as immediate urological complications resulting from FGM [14]. Urine retention usually occurs as immediate complication to FGM because of pain, infection and obstruction of the external urethral meatus by skin flaps or blood clots. However, in this case we highlight a late urological complication of FGM. Also as *Dirie* and *Lindmark* explained the urological symptoms in FGM patients ‘’ urine retention occurred because the meatus usually is covered by the infundibulum, causing the vaginal discharge to accumulate and favor the growth of bacteria. In this case, the patient’s midwife just have re- assured her, thus, it is important that health professionals should be aware of the complications of FGM and recognize its seriousness.

## Conclusion

This case report indicates that FGM is a serious public health problem and there should an urgent intervention such as planned health education campaigns to end FGM practice.

## Data Availability

Data sharing is not applicable to this article as no datasets were generated or. analysed during this study.

## Abbreviations

BOO: Bladder outlet obstruction
cm: Centimeter
FGM/C: Female genital mutilation/cutting
Kg: Kilogram
